# Immunoregulatory Functions of Interferons During Genital HSV-2 Infection

**DOI:** 10.3389/fimmu.2021.724618

**Published:** 2021-08-18

**Authors:** Emily Feng, Elizabeth Balint, Fatemah Vahedi, Ali A. Ashkar

**Affiliations:** McMaster Immunology Research Centre, Department of Medicine, McMaster University, Hamilton, ON, Canada

**Keywords:** HSV – 2, immune regulation, female reproductive tract (FRT), type I interferon (IFN), type II interferon, type III interferons, genital mucosa

## Abstract

Herpes simplex virus type 2 (HSV-2) infection is one of the most prevalent sexually transmitted infections that disproportionately impacts women worldwide. Currently, there are no vaccines or curative treatments, resulting in life-long infection. The mucosal environment of the female reproductive tract (FRT) is home to a complex array of local immune defenses that must be carefully coordinated to protect against genital HSV-2 infection, while preventing excessive inflammation to prevent disease symptoms. Crucial to the defense against HSV-2 infection in the FRT are three classes of highly related and integrated cytokines, type I, II, and III interferons (IFN). These three classes of cytokines control HSV-2 infection and reduce tissue damage through a combination of directly inhibiting viral replication, as well as regulating the function of resident immune cells. In this review, we will examine how interferons are induced and their critical role in how they shape the local immune response to HSV-2 infection in the FRT.

## Introduction

Genital herpes simplex virus type 2 (HSV-2) infection remains one of the most prevalent sexually transmitted infections, with an estimated 491.6 million cases worldwide (1). African cohorts comprise of the majority of the cases, with 43.9% and 25.4% seropositivity in women and men respectively, and HSV-2 disproportionately impacts women in African cohorts and globally (1). Despite the prevalence of HSV-2 infection, no preventative or curative treatments have been developed. Current treatments for HSV-2 infection only involve antivirals to suppress reactivation, but HSV-2 remains a life-long infection. Unfortunately, resistance to antivirals such as acyclovir, particularly in immunocompromised individuals, has been observed (2).

Genital HSV-2 infection in the vaginal mucosa begins at lytic replication in keratinocytes of the epithelial lining, resulting in genital lesions (3). HSV-2 is also a neurotropic virus and can infect the brain and result in fatal herpes simplex encephalitis in newborns or immunocompromised individuals (4, 5). Largely, HSV-2 infections are self-limiting, and are often asymptomatic. However, asymptomatic individuals still frequently shed the virus during reactivation periods (6–8). Those that experience symptomatic infection will often experience reactivation of genital lesions throughout their lifetime (9). While antivirals, such as acyclovir, can reduce the frequency of HSV reactivation, they cannot eliminate or completely prevent viral shedding (9). Furthermore, individuals with genital HSV-2 infection also hold a significantly increased risk of acquiring HIV-1 infection, partially through impaired integrity of the vaginal mucosal barrier due to genital lesions (10, 11). Thus, the inability to adequately address the HSV-2 epidemic has also been driving the HIV epidemic.

Regulation of innate and adaptive immune responses in the genital mucosa is critical in both the control of HSV-2 infection and reactivation, as well as inhibiting the development of genital and neural tissue pathology. Interferons (IFN), including classes I, II, and III, are well known for their antiviral functions, but their role in immune regulation is slowly coming to light. The critical importance of IFNs is best evidenced by the multiple mechanisms of evasion of IFN-induced responses by the HSV-2 virion to evade IFN induction and signaling pathways (12). Understanding how immunity to HSV-2 infections is regulated by IFNs remains a crucial piece in developing better therapeutics and an effective vaccine for genital herpes infections. In this review, we will describe the various mechanisms of IFN-mediated regulation of innate and adaptive immunity to genital HSV-2 infection, emphasizing its ability to prevent the development of pathogenic immunity.

## Induction and Regulation of IFN Responses to Genital HSV-2 Infection

Type I IFNs are a class of signaling molecules that include most notably IFN-α and its subtypes, IFN-β, as well as lesser understood IFN-ϵ, IFN-ω, and IFN-κ (13). The induction of type I IFNs during genital HSV-2 infection contributes significantly to resistance to infection, through both directly suppressing viral replication and facilitating antiviral immune responses (14, 15). Type I IFNs signal through their dimeric interferon α/β receptor (IFNAR) to induce a JAK/STAT signaling cascade that includes the transcription of interferon-stimulated genes (ISGs) to inhibit viral replication (13). Induction of type I IFN occurs through DNA sensing pattern recognition receptors, including endosomal toll like receptor 9 (TLR9), and cytosolic sensors IFI16 and cyclic GMP-AMP synthase (cGAS) stimulating the STING adaptor protein (16, 17). Interestingly, Eriksson et al. demonstrated that polymorphisms in IFI16 impacting expression levels correlated with both IFN-β production and susceptibility to genital HSV-2 infection (18). The administration of both TLR agonists and STING agonists in mice have demonstrated to protect against genital herpes infection and potently induce type I IFNs (14, 19). Recognition of replication intermediate dsRNA has been shown by retinoic acid-inducible gene-like receptors (RLRs) RIG-I and MDA5 (16, 17). IFN-ϵ, produced at canonically low levels in the reproductive mucosa, is not induced through typical TLR or RIG-I signaling pathways (20).

IFN-α/β is primarily produced by circulating plasmacytoid dendritic cells (pDCs) through recognition by TLR9 during genital HSV-2 infection (16, 21, 22). Genital lesions in recurrent HSV-2 patients display pDC infiltration, suggesting a role of type I IFNs in controlling HSV-2 reactivation (23). However, others have demonstrated that pDCs are only involved in systemic infections in mice, and not local mucosal infections (24). These studies have also credited classical CD8α DCs as a major source of IFN production, independent of TLR9 signaling (22, 24). On the other hand, unlike IFN-α/β, IFN-ϵ was found to be constitutively expressed by epithelial cells in the uterus and ovaries of both mice and humans (20). Together, these studies show that the production of type I IFNs is critical to inducing protective innate and adaptive immune responses in response to genital HSV-2 infections.

Type II IFNs consist only of one subtype, IFN-γ. The induction of IFN-γ is strongly dependent on type I IFN signaling. Type II IFNs are largely produced from NK cells and T cells during genital HSV-2 infection, stimulated by type I IFN-mediated IL-18 signaling (25). IFN-γ signaling occurs through the IFN-γ receptor present on the majority of immune cells (13). Several studies have identified a critical role of IFN-γ in mediating protection against genital HSV-2 infection. *In vitro* HSV-2 infection of human PBMCs demonstrates IFN-γ as the predominant cytokine produced, and dysregulated IFN-γ production is associated with recurrent genital herpes in humans (26, 27). This dysregulation has been associated with genetic variations in the *STAT4* gene, which regulates IFN-γ production (28). Individuals with recurrent disease also exhibit impaired cellular responses to IFN-γ signaling, as treatment of HSV-2-infected macrophages from these individuals with IFN-γ enhanced viral replication (26). Likewise, several mouse models have demonstrated that IFN-γ is required for HSV-2 clearance during primary and secondary challenge (28–33).

Type III IFNs have emerged as another family of IFNs with a critical role in the induction of antiviral responses to HSV-2 infection. Type III IFNs, consisting of IFN-λ1, 2, 3, and 4 in humans, and functional IFN-λ2 and 3 in mice, have already been established as potent inhibitors of viral replication (34, 35). DCs and pDCs are a primary producer of type III IFNs in response to TLR7 ligands and HSV-2 infection (36–38). However, most cells can produce type III IFNs, including mucosal epithelial cells. Type III IFNs are similar to the type I IFN canonical signaling pathway, but differ in their effector cells (35, 39). The expression of the IFN-λ heterodimeric receptor, IL-28Rα/IL-10Rβ, is more restricted than IFNAR, as it is highly expressed on mucosal epithelial cells in the genital mucosa (40–43). Some evidence suggests that the type III IFN receptor is not expressed on the surfaces of immune cells, leading to its less inflammatory responses (44); however, this has been increasingly challenged, with emerging evidence suggesting type III IFNs may possess direct immunoregulatory functions.

Susceptibility to HSV-2 infection has been suggested to be influenced by hormone-mediated alterations in IFN responses. In mice, susceptibility to intravaginal HSV-2 infection is dependent on treatment with progesterone contraceptive hormones to induce diestrus, a state of low estrogen levels (45). Estradiol treatment in women increased capacity to produce type I IFN by pDCs following just one month of treatment (46). IFN-ϵ is potentially even more strongly regulated by hormone levels, with lowest levels during diestrus and highest production during estrus, and IFN-ϵ deficient mice display increased susceptibility to HSV-2 infection (20). Meanwhile, progesterone signaling not only increases susceptibility to mouse HSV-2 infection, but impedes type I IFN responses (45, 47, 48). Depot medroxyprogesterone acetate (DMPA) treatment impairs TLR9 ligand-mediated IFN-α production by inhibiting IRF7 nuclear accumulation following CpG stimulation in both human and mouse pDCs (47, 49). These findings strongly emphasize the importance of understanding the impact of hormones, from either biological factors or from contraceptives, on the development of protective immunity to HSV-2 infection, and its considerations on therapeutic development. Moreover, this highlights the dependency on type I IFNs for protection against HSV-2 infection.

## The Role of Interferons in Regulation of Innate Immunity During HSV-2 Infection

Innate immunity in the genital mucosa plays a critical role in initial viral infection and replication, however their dysregulation can also be the cause of severe inflammation and tissue damage. In this section, we will explore the innate immune responses regulated by IFNs towards genital HSV-2 infection.

### Monocytes/Macrophages

During HSV-2 infection, monocyte recruitment is crucial for controlling viral infection and inducing antiviral immunity in the vaginal mucosa (50). Monocytes/macrophages upregulate the expression of Fas and FasL during infection (51). Although typically involved in inducing the apoptosis of infected cells, HSV-2 infected keratinocytes in a mouse model displayed resistance to Fas/FasL-mediated apoptosis, but strong production of inflammatory cytokines, including TNF-α, IL-1β, and CXCL1/2 (52). Infected monocytes in mice, while susceptible to Fas-mediated apoptosis, also respond with the production of inflammatory cytokines and CXCL9/10 T cell chemokines (51).

Evidence has suggested that type I IFN signaling may regulate the protective effects of monocyte/macrophages during HSV-2 infection. Type I IFN has been demonstrated to induce FasL expression during influenza infection in mice on immune cells (53), though this has not been defined during HSV-2 genital infection. Type I IFN regulation of Fas/FasL pathways during HSV-2 infection would promote monocyte-mediated inflammation and the induction and recruitment of adaptive immune responses. Type I IFN-mediated recruitment of inflammatory monocytes (IM) during infection has been clearly defined in murine HSV-1 and HSV-2 infection, promoting survival and antiviral responses (50, 54). The immune stimulatory effect on monocytes is dependent on type I IFN-mediated production of CCL2 during HSV-2 infection that promotes recruitment to the genital mucosa (25, 55). Therefore, type I IFN signaling during HSV-2 infection promotes both the recruitment of inflammatory monocytes to the genital mucosa, but also Fas/FasL-induced inflammation in the clearance of infection.

Type II IFNs produced by NK cells and T cells also promote macrophage responses during HSV-2 infection. Macrophage nitric oxide production is stimulated by IFN-γ signaling, and HSV-2-infected mouse macrophages exhibit enhanced nitric oxide release upon IFN-γ stimulation (56). IFN-γ is a Th1 promoting cytokine shown to promote macrophage M1 polarization and production of pro-inflammatory cytokines, as well as upregulation of MHC class II expression to facilitate effective adaptive immune responses in a mouse model of HSV-2 infection (57, 58). Overall, type I and II IFNs facilitate the recruitment and activation of antiviral functions of both IM and macrophages during HSV-2 infection.

### Neutrophils

Neutrophils can play a protective role in HSV-2 infection, but their dysregulation can lead to damaging inflammatory outcomes. Early neutrophil recruitment to the genital mucosa can limit the establishment of HSV-2 infection in mice (59). IL-36γ production by mucosal epithelial cells in HSV-2 infection suppressed viral replication through neutrophil recruitment and expression of neutrophil chemokines CXCL1 and CXCL2 (60). This in turn prevented viral dissemination, and importantly, neuroinvasion. Interestingly, IL-36γ induction has been demonstrated to increase sensitivity to IFN-α/β during HSV-2 infection in mice through increased IFNAR expression on keratinocytes, likely also playing a role in the protective function of IL-36 (61).

However, despite the role of neutrophils in limiting early viral replication, neutrophils have often been demonstrated to cause damaging inflammatory immune responses and are implicated in increasing disease severity during viral infection (62, 63). Similarly, neutrophils have been described to drive liver damage in a murine model for systemic HSV-2 infection (64). These pathogenic effects of neutrophils are strongly regulated by type I IFN signaling. While CXCL1 is upregulated *in vitro* following HSV-2 infection, type I IFN signaling can suppress the expression of neutrophil chemokines by IM and reduce their recruitment to the sensory ganglia during mouse HSV-1 infection and other mucosal infections, such as IAV (62, 65). This process of IFN-mediated regulation of neutrophils would inhibit HSV-2 infection-induced neuronal damage. Similarly, IFN-λ has been shown to suppress neutrophil-mediated damage in mice in the intestinal mucosa, though this has yet to be confirmed in the genital mucosa (66). On the contrary, dysregulated and prolonged type I IFN signaling was recently described to promote epithelial damage in response to mouse HSV infection by neutrophils (67). Nonetheless, early type I IFN signaling in this model is still important for viral control and survival of HSV-2 infection (67). In conclusion, we observe that regulated type I IFN responses during HSV-2 infection promotes protective neutrophil functions while also suppressing pathogenic immune activation in the central nervous system (CNS).

### NK Cells

It has been well established that Natural Killer (NK) and NKT cells are critically required for innate protection against HSV-2 infections, and cases of individuals with severe NK cell deficiencies have been associated with recurrent HSV infections (68). NK cell-deficient mouse models are highly susceptible to HSV-2 infection, with greater viral load and mortality (69). Likewise, loss of NK cell recruitment by chemokines CXCL9 and CXCL10, or loss of the CCR5 receptor, increased susceptibility to intravaginal HSV-2 infection in mice (70, 71). Impairment of NK cell recruitment increased viral load in both the genital mucosa and the central nervous system, highlighting its critical role in suppressing local viral replication as well as neuroinvasion (70, 71).

Type I IFN production during infection is essential for NK cell activation and has long been suggested to activate NK cells (72–75). Furthermore, NK cell memory has been observed in mouse HSV-2 infection, and type I IFN signaling promotes NK cell expansion, protection against fratricide, and induction of NK cell memory (76–78). However, we and others have shown that type I IFNs induce NK cell activation through an indirect mechanism. Loss of IFNAR in a mouse model of HSV-2 infection suppressed IFN-γ production by NK cells, but not NK cell recruitment to vaginal tissue at 2 days post-infection, suggesting a role in NK cell activation but not recruitment (79). In response to systemic and local infection, as well as TLR stimulation, type I IFN signaling on DCs is known to induce IL-15 trans-presentation to NK cells recruited to lymph nodes, as well as increased IL-15Rα expression (79). However, absence of type I IFN signaling in a mouse model of HSV-2 genital infection resulted in enhanced IL-15 production, with no impacts on IL-15Rα expression (25, 72). Thus, impaired NK cell activation in the absence of type I IFN signaling is not mediated by reduced IL-15 trans-presentation.

During HSV-2 infection, IMs are key mediators of NK cell activation. Type I IFN signaling *via* IFNAR on IM induces the production of the cytokine IL-18 typically capable of inducing NK cell IFN-γ production (25). IFN-λ3 may also induce NK cell activity through direct signaling on monocyte-macrophages and their production of NK cell stimulatory cytokines, as seen in influenza infection in mice (80). While IL-12 and IL-18 are well-known to synergize for optimal NK cell IFN-γ production, IM production of IL-18 was critical for NK cell activation in the context of HSV-2 infection (25, 81–83). In *Ifnar^-/-^* and *Il-18^-/-^* mice, reduced IL-18 production resulted in a complete lack of IFN-γ production during early HSV-2 infection (25). This was specifically due to a lack of NK cell activation, as NK cell recruitment was not affected by IFNAR and IL-18 deficiency. Overall, type I IFNs are critical mediators of indirect NK cell activation and IFN-γ production during mouse HSV-2 infection.

In addition to its role in the induction of adaptive immunity and type II IFN production, type I IFN has also been suggested to possess immunoregulatory properties for NK cells. Interestingly, direct type I IFN signaling on both human and mouse NK cells inhibits NK cell IFN-γ production (84). Excessive IFN-γ production has been shown to promote immune-mediated tissue damage. For example, high production of IFN-γ in the central nervous system can lead to microglia-mediated demyelination and neurological sequelae in mice (85, 86). Similarly, IFN-γ production during mouse HSV-2 infection is tightly regulated by type I IFNs to prevent excessive immune activation and immunopathology, peaking at 2 days post-infection (dpi) and rapidly decreasing by 3 dpi (84). Thus, type I IFN signaling during HSV-2 infection critically regulates both the induction and control of NK cell-mediated type II IFN signaling.

## The Role of Interferons in Regulation of Adaptive Immunity During HSV-2 Infection

As we have discussed, induction of effective and appropriate innate immune responses is critical for early protection against HSV-2 infection, as well as restricting immune-mediated damage. However, IFNs are also necessary to direct an appropriate adaptive immune response and develop immune memory. This section will clarify the mechanisms through which IFNs promote Th1 adaptive immune responses and vaccine-induced memory while preventing harmful, suboptimal T and B cell responses.

### Dendritic Cells Mediate Innate/Adaptive Crosstalk

To establish an appropriate adaptive immune response to HSV-2 infection, early antiviral responses *via* type I IFN signaling are required. The FRT consists of various populations of antigen presentating cells (APCs), including Langerhans cells in the epithelium, submucosal DCs, and monocyte-derived DCs (87). As previously described, IFNs are necessary for the recruitment of IM to the vaginal tract. Iijima et al. demonstrated that type I IFN signaling during HSV-2 infection in mice is required to recruit IM *via* CCR2, and that IM-derived DCs play a distinct role in restimulation of effector Th1 CD4^+^ T cells to produce IFN-γ (50). Type I IFNs have also been demonstrated to induce DC maturation and DC-facilitated Th1 responses in mice (88). Submucosal DCs migrating to the draining lymph node have been shown to induce Th1 responses to genital HSV-2 infection in mice (89). Furthermore, both monocyte-derived DCs and submucosal lamina propria DCs stimulate the protective responses of memory CD8+ T cells established by HSV-2 333 TK- immunization in mice (33, 50). Thus, type I IFNs promote both local and systemic DC-facilitated Th1 immune responses during HSV-2 infection.

Similarly, IFN-γ plays a critical role in stimulating APC functions for the development of adaptive immunity. Activation of macrophages and upregulation of epithelial MHC class II expression by IFN-γ signaling contributes to the development of effective T cell immunity (57, 58). Similarly, the kinetic differences in IFN-γ production between genital HSV-1 and HSV-2 infection in mice define their pathological outcomes, as early NK cell-derived IFN-γ production following genital HSV-1 infection induced rapid DC maturation and migration to the draining lymph node to protect against neuroinvasion (90). However, genital HSV-2 infection in mice did not elicit the same early burst of IFN-γ production and resulted in greater nervous system infection and more severe disease (90). IFN-γ signaling further promotes T cell responses by regulating the expression of costimulatory molecule B7 isoforms on APCs (26). However, IFN-γ treatment of human PBMCs failed to enhance B7-1 or B7-2 expression on monocytes from individuals with recurrent HSV-2 infection, which may explain impaired T cell-mediated immunity and viral clearance in these individuals (26). Overall, these studies demonstrate how inducing a potent and early IFN-γ response is critical in APC function and mounting an effective and protective adaptive immune response to HSV-2.

### T Cells

CD8^+^ T cells provide fundamental antiviral protection by perforin and granzyme-mediated lysis of infected cells as well as production of the type II IFN, IFN-γ (31). Additionally, IFN-γ production by T cells is an essential component of the memory response to HSV-2 reinfection, as HSV-2 333TK^—^immunized CD4^-/-^ mice cannot clear genital HSV-2 infections and do not survive lethal HSV-2 challenge (32, 91). This protection during reinfection is mediated by CD4^+^ T cell-derived IFN-γ as well as their ability to orchestrate enhanced NK cell activation and IFN-γ production (92). Further vaccination studies eliciting tissue resident memory CD8^+^ T cells suggest IFN-γ is required for vaccination-induced protection against HSV-2 (33).

Type I IFNs promote T cell effector function indirectly *via* APCs, as described in section 4.1. Additionally, type I IFNs have been shown to directly act on mouse T cells to promote expansion, memory formation, and effector functions that are critical for HSV-2 clearance (93–96). However, type I IFNs may also possess immunoregulatory functions to prevent excessive T cell activation and subsequent tissue damage. High levels of type I IFN signaling through Poly(I:C) and IFN-α/β treatment *in vivo* has been seen to induce attrition or reduced proliferation of CD8^+^ T cells (97–99). Thus, an appropriate type I IFN response is critical to balance activation and regulation of T cell responses during HSV-2 infection.

As we have previously outlined, type I IFNs critically regulate NK cell and T cell-derived production of IFN-γ. The development of effective T cell responses to HSV-2 infection is also dependent on IFN-γ. IFN-γ stimulates chemokine production for the recruitment of immune cells, as mouse HSV-2 vaccination has been associated with induction of local IFN-γ-dependent RANTES production (100). Similarly, HSV-2 vaccination in mice can also facilitate retention of vaginal tissue resident memory CD4^+^ T cells by IFN-γ-dependent macrophage production of CCL5 and CXCL9 (101). As seen above, IFN-γ promotes APC maturation and facilitation of Th1 responses to HSV-2 infection. In contrast, dysregulation or deficiency of IFN-γ in HSV-2-infected individuals and mice has been observed to promote a Th2 cytokine response through IL-10 in humans and IL-4 in mice, which is unable to facilitate viral clearance (26, 91). Th2 cytokine driven immune responses to vaginal HSV-2 infection in mice have also been described as pathogenic (102). Thus, type II IFNs are critical mediators of T cell-mediated viral clearance, and prevent pathogenic Th2 immunity during primary and secondary HSV-2 infection. An effective vaccine against HSV-2 must elicit potent T cell-mediated IFN-γ release for optimal protection against HSV-2 infection.

### B Cells

A role for B cell-mediated innate protection against HSV-2 infection has been described, as B cell-deficient mice exhibited transient infection and inflammation, while T cell-deficient mice did not exhibit infection-induced inflammation (32). Passive transfer of serum from uninfected mice reduced vaginal HSV-2 titers, suggesting innate protection by natural antibodies. Thus, the sheer presence of antibodies in the vaginal mucosa contributes to innate-like protection against HSV-2 infection. Further, naïve B cells and antibody-secreting cells have recently been identified in recurrent HSV-2 lesions alongside CD4^+^ T cells, suggesting a role for B cells in the resolution of reactivated HSV-2 lesions (103).

B cells also contribute to protective memory responses against HSV-2 reinfection following immunization with HSV-2 333 TK^-^ and live-attenuated 0ΔNLS strains of HSV-2 (104, 105). Several studies have shown that type I IFNs directly and indirectly regulate B cell proliferation, plasma cell differentiation, and isotype switching (93, 106–109). Thus, type I IFN signaling promotes induction of memory B cell development and vaccine efficacy. Likewise, type II IFN responses are fundamental for B cell recruitment, priming, and function during primary and secondary HSV-2 infection. Upon secondary challenge, IFN-γ was shown to mediate the production of chemokines, such as CXCL9 and CXCL10, that recruit memory B cells that are capable of rapid antibody secretion (110). CD4^+^ T cell IFN-γ production following immunization also plays an important role in antibody access to neuronal tissues to enable viral control (111). In another study, IFN-γ deficiency did not impact overall serum IgG levels in TK^–^ immunized mice (91). Instead, they found that IgG1 antibodies were enriched in IFN-γ^-/-^ mice, whereas IgG2 was favoured in WT mice. Although this did not significantly influence early protection against HSV-2, these IgG1 antibodies were less effective in preventing HSV-2 spread to the nervous system (91). This suggests that dysregulation of IFN-γ, as observed in individuals with recurrent HSV-2 infection (26, 28), may support viral entry into the CNS and the establishment of latent infection *via* altered isotype switching. Thus, IFN-γ is critically required to recruit and promote optimal B cell responses, while preventing suboptimal isotype switching, to protect against HSV-2 infection.

### The Role of IFNs in HSV-2 Vaccine Development

Mouse models of HSV-2 vaccination have largely demonstrated potent memory T cell and neutralizing antibody responses capable of protection following subsequent HSV-2 challenge (29, 32, 33, 91, 92, 100, 111). However, these preclinical results have not translated to vaccine efficacy in humans during clinical trials. Several candidate vaccines have exhibited poor efficacy in clinical trials with varying induction of neutralizing antibody or cellular responses (112–117). Although vaccination models in mice demonstrate the requirement for memory CD4^+^ T cell-derived IFN-γ for optimal protection, most clinical studies in humans focus on neutralizing antibody titer and provide little to no examination of CD4^+^ T cell specificity or IFN-γ production (113, 116–118).

Although type I IFNs may not be required for vaccine-induced memory responses in mice (119), HSV-2 evasion of type I IFN signaling, demonstrated by a lack of type I IFN in human lesion biopsies compared to mice, may reduce the efficacy of vaccine-induced memory responses (120). As evidenced by multiple clinical trial failures despite successful pre-clinical models, the factors behind the development of protective adaptive immunity to genital HSV-2 infection differ between human and animal models. In addition, vaccination studies in mice do not consider the role of type I IFN in regard to the longevity of the established protective immunity, with studies administering secondary challenge under a month post-vaccination (119). The role of type III IFNs should also be carefully considered, as the use of IFN-λ3 as an adjuvant in a mouse vaccination model enhanced both humoral and cellular immune responses, resulting in improved vaccine efficacy (121). Thus, a lack of type I, II, and III IFN induction by HSV-2 vaccination in humans may prevent the development of protective adaptive immunity and should be carefully considered in future vaccine development.

## Concluding Remarks

**Figure 1:**
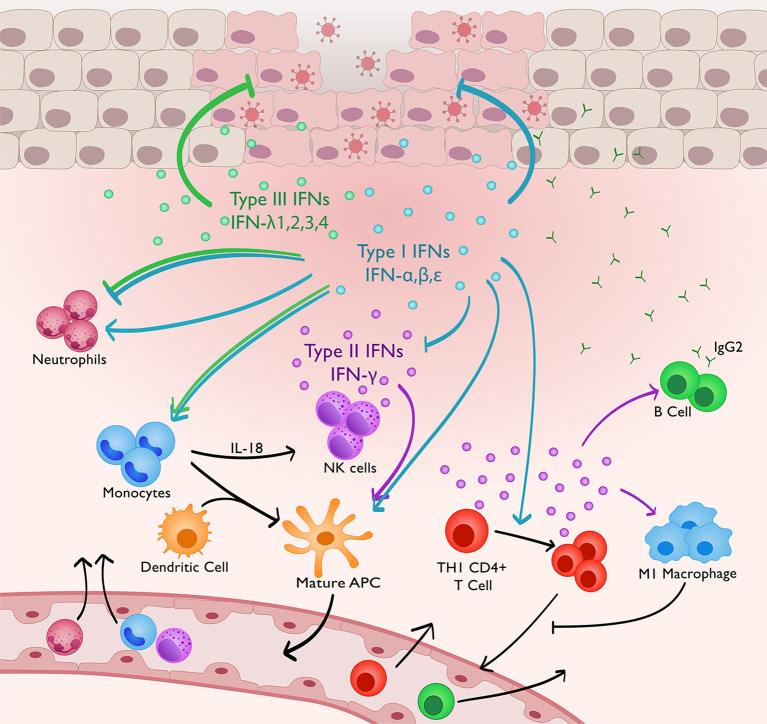
IFNs regulating innate and adaptive immunity to genital HSV-2 infection. Type I and III IFNs directly suppress HSV-2 replication in the genital mucosa. Concurrently, type I IFN signaling promotes IM recruitment and production of IL-18. This in turns stimulates NK cell IFN-γ production. Type I IFNs stimulate adaptive immunity both directly and indirectly through IM differentiation into APCs, maturation of DCs, and inducing CD4+ T cell expansion. Type I IFNs can both promote neutrophil function, and negatively regulate innate immune responses to prevent immune-mediated pathology by suppressing neutrophil recruitment and limiting IFN-γ production by NK cells. IFN-γ production, highly dependent on type I IFN signaling, promotes IgG2 antibody production, M1 macrophage polarisation and DC maturation, which will promote CD4+ memory T cell retention *via* production of CCL4 and CXCL9. Type III IFNs, similar to type I IFNs, have also been shown to suppress neutrophil activity and monocyte-mediated NK cell activation, but this is not yet demonstrated in the vaginal mucosa.

The development of novel preventative and prophylactic treatments to sexually transmitted HSV-2 has been hindered by an inability to induce a strong, protective immune response in the genital mucosa. As summarized in **Figure 1**, the role of IFNs extends beyond suppressing viral replication, but also in the development of local protective innate and adaptive immune responses to viral infection. Type I and II IFN signaling directly, and indirectly through the induction of innate immunity, regulates the development of immune memory and adaptive responses to viral infection. Similarly, type III IFNs also play a critical role in controlling viral replication, and likely have many additional unexplored functions in regulating innate immune responses in the genital mucosa. Impaired type I IFN signaling during genital herpes infection will consequently impair type II IFN responses, which aid in the development of adaptive immunity and are critical for HSV-2 clearance. As previously described, dysregulated responses to IFN-γ are observed in recurrent HSV-2-infected individuals (26). Future treatments or vaccines must restore responsiveness to IFN-γ signaling and elicit potent CD4^+^ T cell-derived IFN-γ production due to its multifaceted role in immune regulation and antiviral immunity.

As exemplified by this review, proper induction of type I, II, and III IFNs is critical for the development of immunity and protective memory against genital HSV-2 infections. The use of IFNs as a therapeutic strategy against HSV-2 could provide a promising avenue. In addition, a greater research focus on the role of type III IFNs should be placed, including both their immunoregulatory functions and their potential as a treatment against shedding and reactivation. While type I IFNs offer invaluable protection during the early stages of infection, their presence can also promote pathogenic immune responses during mucosal infection (67, 122). Type III IFN treatment of various other viral infections, such as with SARS-CoV-2 patients and an IAV mouse model, have demonstrated it to be both safe and effective (123, 124). Overall, both the antiviral and regulatory functions of IFNs in vaginal HSV-2 infection must be carefully and seriously considered in the development of novel therapeutics, prophylactic treatments, and vaccines.

## Author Contributions

EF and EB conceptualised the review. EF, EB, and FV were responsible for writing the manuscript. EF, EB and AA edited the manuscript. AA supervised and guided the development of the manuscript. All authors contributed to the article and approved the submitted version.

## Funding

This work was supported by the Canadian Institutes of Health Research (CIHR). AA is also a recipient of a CIHR Tier 1 Canada Research Chair. EF is supported by the Ontario Graduate Scholarship. EB is a recipient of the CIHR Canada Graduate Scholarship-Master’s.

## Conflict of Interest

The authors declare that the research was conducted in the absence of any commercial or financial relationships that could be construed as a potential conflict of interest.

## Publisher’s Note

All claims expressed in this article are solely those of the authors and do not necessarily represent those of their affiliated organizations, or those of the publisher, the editors and the reviewers. Any product that may be evaluated in this article, or claim that may be made by its manufacturer, is not guaranteed or endorsed by the publisher.
